# AI-algorithm training and validation for identification of endometrial CD138+ cells in infertility-associated conditions; polycystic ovary syndrome (PCOS) and recurrent implantation failure (RIF)

**DOI:** 10.1016/j.jpi.2024.100380

**Published:** 2024-04-29

**Authors:** Seungbaek Lee, Riikka K. Arffman, Elina K. Komsi, Outi Lindgren, Janette A. Kemppainen, Hanna Metsola, Henna-Riikka Rossi, Anne Ahtikoski, Keiu Kask, Merli Saare, Andres Salumets, Terhi T. Piltonen

**Affiliations:** a Department of Obstetrics and Gynaecology, Medical Research Center Oulu, Research Unit of Clinical Medicine, University of Oulu and Oulu University Hospital, Oulu 90220, Finland; b Department of Obstetrics and Gynaecology, Institute of Clinical Medicine, University of Tartu, Tartu 50406, Estonia; c Department of Pathology, Oulu University Hospital, Cancer and Translational Medicine Research Unit, University of Oulu, Oulu 90220, Finland; d Department of Pathology, Turku University Hospital, Turku 20521, Finland; e Competence Centre on Health Technologies, Tartu 51014, Estonia; f Division of Obstetrics and Gynaecology, Department of Clinical Science, Intervention and Technology, Karolinska Institute and Karolinska University Hospital, Stockholm 14152, Sweden

**Keywords:** Artificial intelligence, CD138, Chronic endometritis, Polycystic ovary syndrome, Recurrent implantation failure, Computational histology

## Abstract

**Background:**

Endometrial CD138+ plasma cells serve as a diagnostic biomarker for endometrial inflammation, and their elevated occurrence correlates positively with adverse pregnancy outcomes. Infertility-related conditions like polycystic ovary syndrome (PCOS) and recurrent implantation failure (RIF) are closely associated with systemic and local chronic inflammatory status, wherein endometrial CD138+ plasma cell accumulation could also contribute to endometrial pathology. Current methods for quantifying CD138+ cells typically involve laborious and time-consuming microscopic assessments of only a few random areas from a slide. These methods have limitations in accurately representing the entire slide and are susceptible to significant biases arising from intra- and interobserver variations. Implementing artificial intelligence (AI) for CD138+ cell identification could enhance the accuracy, reproducibility, and reliability of analysis.

**Methods:**

Here, an AI algorithm was developed to identify CD138+ plasma cells within endometrial tissue. The AI model comprised two layers of convolutional neural networks (CNNs). CNN1 was trained to segment epithelium and stroma across 28,363 mm^2^ (2.56 mm^2^ of epithelium and 24.87 mm^2^ of stroma), while CNN2 was trained to distinguish stromal cells based on CD138 staining, encompassing 7345 cells in the object layers (6942 CD138− cells and 403 CD138+ cells). The training and performance of the AI model were validated by three experienced pathologists. We collected 193 endometrial tissues from healthy controls (*n* = 73), women with PCOS (*n* = 91), and RIF patients (*n* = 29) and compared the CD138+ cell percentages based on cycle phases, ovulation status, and endometrial receptivity utilizing the AI model.

**Results:**

The AI algorithm consistently and reliably distinguished CD138− and CD138+ cells, with total error rates of 6.32% and 3.23%, respectively. During the training validation, there was a complete agreement between the decisions made by the pathologists and the AI algorithm, while the performance validation demonstrated excellent accuracy between the AI and human evaluation methods (intraclass correlation; 0.76, 95% confidence intervals; 0.36–0.93, *p* = 0.002) and a positive correlation (Spearman's rank correlation coefficient: 0.79, *p* < 0.01). In the AI analysis, the AI model revealed higher CD138+ cell percentages in the proliferative phase (PE) endometrium compared to the secretory phase or anovulatory PCOS endometrium, irrespective of PCOS diagnosis. Interestingly, CD138+ percentages differed according to PCOS phenotype in the PE (*p* = 0.03). On the other hand, the receptivity status had no impact on the cell percentages in RIF samples.

**Conclusion:**

Our findings emphasize the potential and accuracy of the AI algorithm in detecting endometrial CD138+ plasma cells, offering distinct advantages over manual inspection, such as rapid analysis of whole slide images, reduction of intra- and interobserver variations, sparing the valuable time of trained specialists, and consistent productivity. This supports the application of AI technology to help clinical decision-making, for example, in understanding endometrial cycle phase-related dynamics, as well as different reproductive disorders.

## Introduction

Fluctuations in sex hormones throughout the menstrual cycle play a crucial role not only in the growth and degeneration of the endometrial lining but also in shaping the local immune environment by regulating innate/adaptive immune responses and endometrial immune cell populations, like B and T lymphocytes, uterine natural killer cells, plasma cells, macrophages, and dendritic cells.^1^, ^2^, ^3^ As a sum effect, cellular immunity in the endometrium is high in the proliferative phase (PE) but declines toward the mid-secretory phase (MSE), facilitating successful implantation of the semi-allogenic embryo.^2^

Endometrial plasma cells can either be recruited from the circulation or developed locally from antigen-committed B cells.^4^ CD138, a heparan sulfate proteoglycan on the surface of plasma cells, serves as a receptor for growth factors and immune mediators attracting plasma cells to the endometrium.^5^ Endometrial CD138+ plasma cells have been used as a diagnostic biomarker for endometrial inflammatory conditions, including chronic endometritis (CE),^6^, ^7^, ^8^ and the number of CD138+ plasma cells is correlated with the severity of endometrial inflammation in women with reproductive failure.^7^, ^8^, ^9^ Detecting isolated CD138+ plasma cell aggregates without other histological features of CE, such as stromal edema and increased stromal cell density, may also identify cases with potentially milder inflammation and related endometrial dysfunction.^10^ However, the current quantification methods of CD138+ cells typically involve laborious and time-consuming microscopic assessments of only a few randomly selected areas from a tissue slide.^10^ These methods have limitations in accurately representing the entire slide and are prone to significant biases arising from both intra- and interobserver variations.^10^, ^11^, ^12^

Convolutional neural networks (CNNs) consist of multiple layers that operate in a feedforward manner between the layers.^13^ In digital pathology, CNN, the most widely used deep learning algorithm, is highly effective for analyzing histological patterns.^13^ In particular, the artificial intelligence (AI) embedded in CNNs allows for the rapid analysis of whole tissue slides with a high resolution, overcoming the current limitations of manual histopathological performance.^13^ This promising and powerful AI technique has increasingly been applied in various clinical research fields, predicting the onset and progression of diseases.^14^, ^15^, ^16^, ^17^, ^18^ Indeed, we have recently demonstrated the excellent power and accuracy of an AI to identify endometrial epithelium compartments from the stroma in our previous study.^19^ Given that an imbalanced inflammatory milieu has been linked to infertility-associated conditions, such as polycystic ovary syndrome (PCOS)^20^ and recurrent implantation failure (RIF),^9^^,^^10^ our study leverages an AI algorithm to evaluate endometrial CD138+ cells in these specific conditions.

PCOS is a common endocrine disorder characterized by hyperandrogenism (HA), systemic low-grade inflammation, insulin resistance, and irregular menstrual cycles.^21^ The PCOS endometrium exhibits dysregulated immune profiles^20^ and altered receptivity-related proteomic profiles, which could lead to poor reproductive outcomes.^20^^,^^22^ However, many studies lack adequately dated endometrial samples, often overlooking secretory phase (SE) samples despite occasional spontaneous ovulations and pregnancies in PCOS women. Moreover, while the presence of CD138+ cells varies with the menstrual cycle phase,^8^^,^^12^ CD138+-based evaluation in PCOS endometrium across different cycle phases is lacking.

RIF, often defined as three or more failed in vitro fertilization (IVF) attempts with good-quality embryos transferred, significantly impacts IVF success rates.^23^ Maternal factors, such as age, body mass index (BMI), and immunological factors,^23^ as well as unprepared endometrium for embryo implantation (e.g., shifted window of implantation (WOI), impaired decidualization in stroma),^24^^,^^25^ influence the implantation rate and reproductive outcomes.^23^^,^^26^ Additionally, CE is prevalent in RIF patients, affecting up to 14% of this population.^9^ Given the multifaceted pathology of RIF, it is important to understand the association between endometrial receptivity and inflammatory processes, particularly CD138+ cell clustering in fully receptive samples without prominent CE.

In this study, we developed an AI algorithm to identify stromal CD138+ cells, serving as an indicator of the endometrial immune milieu. As a novel setup, we examined the occurrence of CD138+ cells in relation to menstrual cycle phases and ovulatory status in the context of PCOS conditions, as well as endometrial receptivity in cases of RIF, to uncover factors influencing their aggregation.

## Materials and methods

### Tissue collection

#### PCOS and control samples

**Fig. 1:**
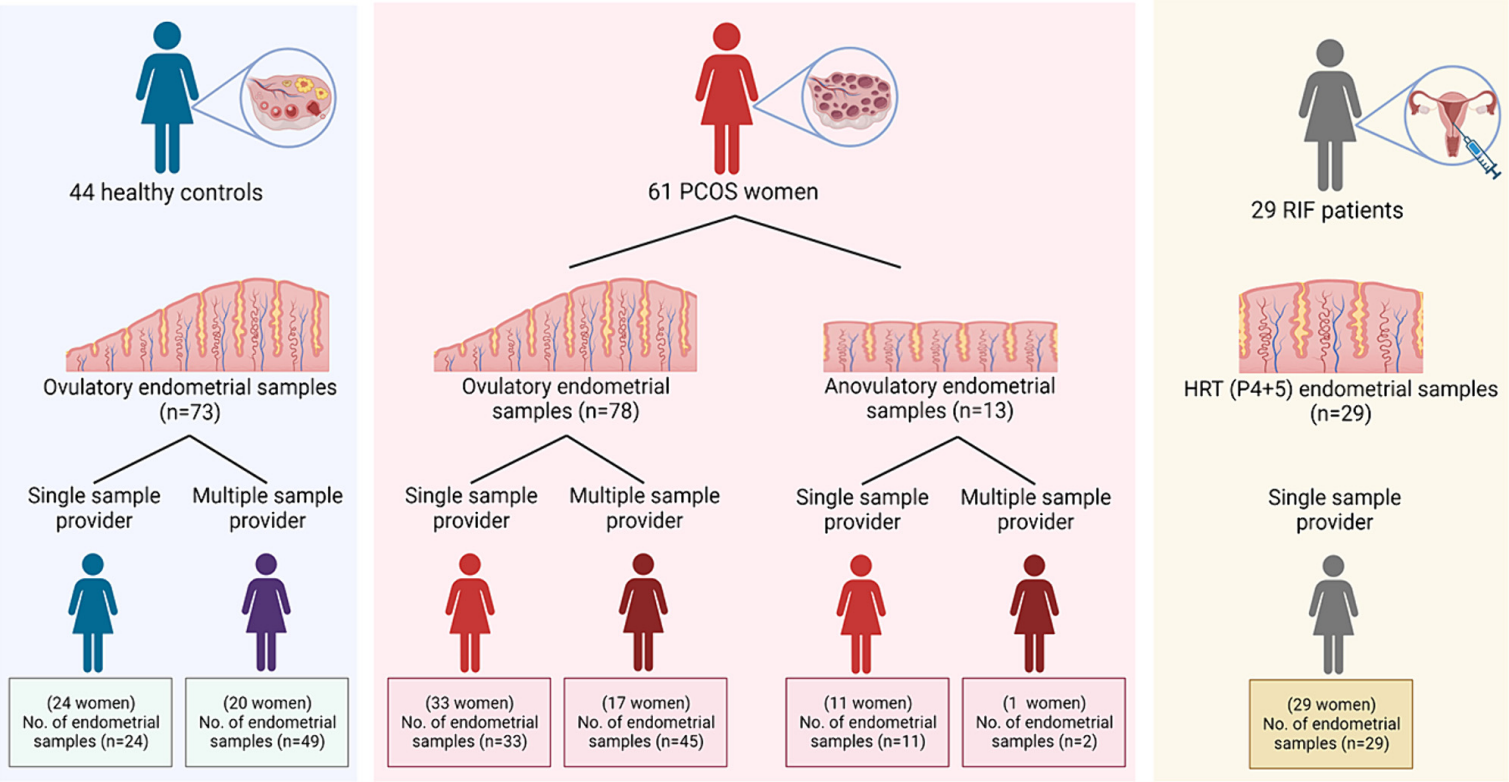
The study subjects in the control, PCOS, and RIF population. A total of 164 endometrial biopsy samples were collected from 44 healthy controls, 61 women with PCOS, and 29 RIF patients. The subjects were categorized based on whether they provided a single or multiple samples. In the case of women with PCOS, the subjects were additionally classified based on their ovulatory status. HRT (hormone replacement treatment); P4 (progesterone).

A total of 164 endometrial biopsy samples from 44 healthy controls and 61 women with PCOS were collected at the Oulu University Hospital (Oulu, Finland) from January 2017 to March 2020 (Fig. 1). The study was approved by The Regional Ethics Committee of the Northern Ostrobothnia Hospital District, Finland (65/2017), and all study subjects signed informed consent. The study was done in accordance with relevant guidelines/regulations in line with the Declaration of Helsinki. All women were non-smokers and had not used any hormonal medication for at least 3 months prior to tissue collection. Some women gave samples in multiple phases in different cycles (only one biopsy per cycle), with a maximum of three samples per individual (depicted by multiple sample providers in Fig. 1). Control women were healthy with regular cycles and had no PCOS symptoms. As per the international guideline, PCOS was diagnosed according to the Rotterdam consensus, requiring the presence of at least two of the following clinical features: oligo-anovulation (OA), HA, and polycystic ovarian morphology (PCOM).^27^ Sampling and analysis were conducted throughout the menstrual cycle in women with PCOS who had occasional ovulations. As for PCOS phenotype sub-analysis, PCOS women were divided based on their phenotype; 35 women with phenotype A (PCOM + OA + HA, most severe PCOS phenotype) and 19 with phenotype D (PCOM+OA, mildest phenotype) were identified (Supplemental Table 1).^27^ Endometrial biopsies were obtained by a suction curette (Pipelle), fixed in 10% formaldehyde for hematoxylin and eosin (H&E) staining and immunohistochemistry (IHC). The samples for ovulatory cycles were obtained in either the PE (cycle days 6–8) or the SE on specific days after the luteinizing hormone (LH) surge, at +2–4 days (early secretory phase (ESE)), +7–8 days (MSE), or +11–12 days (late secretory phase (LSE)) (PE, 12 control, 24 PCOS; ESE, 15 control, 15 PCOS; MSE, 26 control, 21 PCOS; LSE, 20 control, 18 PCOS) (Table 1). The LH surge was detected using a Clearblue digital urine test in the morning, and the presence of the corpus luteum was confirmed by transvaginal ultrasonography (TVUS) via Voluson E8 (GE Healthcare Technologies, United States). The cycle phases were histologically determined from the H&E-stained slides by an experienced gyneco-pathologist. In the case of anovulation confirmed by clinical evaluation and later histological analysis, 13 biopsies were obtained on any day convenient for the PCOS subjects.

**Table 1:** Subject and sample information of age and BMI in different cycle phases and receptivity status.

	Cycle	*N*	Age	*p*-val; Cycle phases^a^	*p*-val; Control vs. PCOS^b^	N	BMI	*p*-val; Cycle phases^a^	*p*-val; Control vs. PCOS^b^
Control ovulatory samples	PE	12	32.42±3.85	0.99		12	28.51±4.83	0.24	
	ESE	15	32.40±6.31			15	26.87±4.43		
	MSE	26	32.08±5.98			26	25.38±4.69		
	LSE	20	31.95±6.09			20	27.49±5.17		
*PCOS*									
Ovulatory samples	PE	24	33.25±4.91	0.80	0.61	24	29.08±5.92	0.13	0.77
	ESE	15	34.64±3.59		0.44	15	29.09±5.98		0.27
	MSE	21	34.14±4.52		0.20	21	25.46±4.58		0.81
	LSE	18	34.11±4.19		0.22	18	28.46±6.05		0.54
Anovulatory samples	No	13	29.77±5.54	**0.01**	**0.05**	13	30.71±9.08	0.32	0.21

RIF	Receptivity	*N*	Age	*p*-val; Receptivity^a^		N	BMI	*p*-val; Receptivity^a^	

Receptivity samples	Pre	9	38.67±5.34	0.97		9	25.68±5.47	0.87	
	Re	9	39.00±5.15			10	25.87±6.02		
	Post	10	37.70±6.85			10	25.47±2.87		

Age and BMI were presented as mean±standard deviation. The statistical differences were calculated by Mann–Whitney *U* test for comparing two groups and Kruskal–Wallis test for multiple groups. There was one missing age data point for a receptive RIF woman.

BMI (body mass index), PE (proliferative phase), ESE (early secretory phase), MSE (mid-secretory phase), LSE (late secretory phase), No (no cycle), Pre (pre-receptive), Re (receptive), Post (post-receptive).

a The statistical analysis between: (i) cycle phases within the control or PCOS ovulatory group, (ii) PCOS ovulatory and anovulatory groups, or (iii) receptivity status within the RIF group.

b The statistical analysis between: (i) control and PCOS ovulatory groups at the same cycle phases and (ii) control, PCOS ovulatory, and anovulatory groups.

Endometrial thickness was measured by TVUS. Serum levels of anti-Müllerian hormone (AMH), LH, follicle-stimulating hormone (FSH), and sex hormone-binding globulin (SHBG) were measured with Elecsys assays (Roche) using a cobas e411 analyzer. Serum testosterone and progesterone (P4) levels were measured at the University of Eastern Finland using an Agilent 1290 Rapid Resolution LC System (Agilent, San Jose, CA, United States).^28^ Free androgen index (FAI) was calculated using the formula: testosterone (nmol/L) / SHBG (nmol/L) *100.

#### RIF samples

A total of 29 RIF samples were obtained from the beREADY endometrial receptivity testing laboratory (CCHT, Tartu, Estonia) from women who had undergone an average of 3.9 unsuccessful IVF cycles despite having good-quality embryos transferred (Fig. 1). The study was approved by the Research Ethics Committee of the University of Tartu, Estonia (340T-12), which also waived the need for informed consent to use the RIF samples, as the data were anonymized. The data and endometrial biopsies were obtained prior to the subsequent IVF treatment^24^ using a Pipelle during a hormone replacement treatment (HRT) cycle on day 5 after initiation of P4 administration (HRT: P + 5). The first day of P4 administration was considered day zero (HRT: P + 0). All biopsies were placed into RNAlater (Ambion, United States) and stored at −80 °C.

### Endometrial receptivity testing

Gene expression profiling was conducted using the beREADY test at CCHT, involving 57 known endometrial receptivity genes along with four housekeeping genes (*SDHA*, *CYC1*, *TBP*, and *HMBS*).^24^^,^^25^ Endometrial RNA from RIF patients (*n* = 29) was extracted using the Qiagen miRNeasy Mini kit, and according to the test results, the RIF samples were divided into pre-receptive (*n* = 9), receptive (*n* = 10), and post-receptive (*n* = 10) (Table 1).

### Immunohistochemistry (IHC)

The endometrial biopsies were processed accordingly: 5-μm paraffin-embedded tissue sections of controls (*n* = 73) and women with PCOS (*n* = 91) and 2.5-μm paraffin sections of RIF patients (*n* = 29) were de-paraffinized in xylene and rehydrated in different grades of alcohol. Next, antigen retrieval with Tris-EDTA (pH 9) was performed in a microwave oven (800 W) for 2 min. Dako peroxidase blocking solution (Dako S2023) was used to neutralize endogenous peroxidase for 5 min. The sections were incubated with 40× diluted mouse anti-human monoclonal antibody CD138 (MS-1793-S; Thermo Fisher Scientific) for 30 min at room temperature and then for 30 min with Dako EnVision polymer (Dako K5007), followed by a DAB working solution for 3 min. H&E staining was performed for 15 s. The IHC staining for both controls and PCOS samples was performed at the University of Oulu (Oulu, Finland), and the staining for the RIF samples at the Tartu University Hospital Pathology Department and the University of Tartu (Tartu, Estonia). Positive and negative controls from four different human tissues (i.e., tonsils, liver, pancreas, and appendix) were used for the CD138 antibody.

All IHC slides were scanned using a Leica SCN 400 Slide Scanner (Leica, Biosystems, United States), and the digitalized whole-slide images (WSIs) were uploaded to Aiforia cloud (Aiforia Technologies Oy, Helsinki, Finland). Eleven slides with inadequate quality were excluded.

### AI algorithm training

**Fig. 2:**
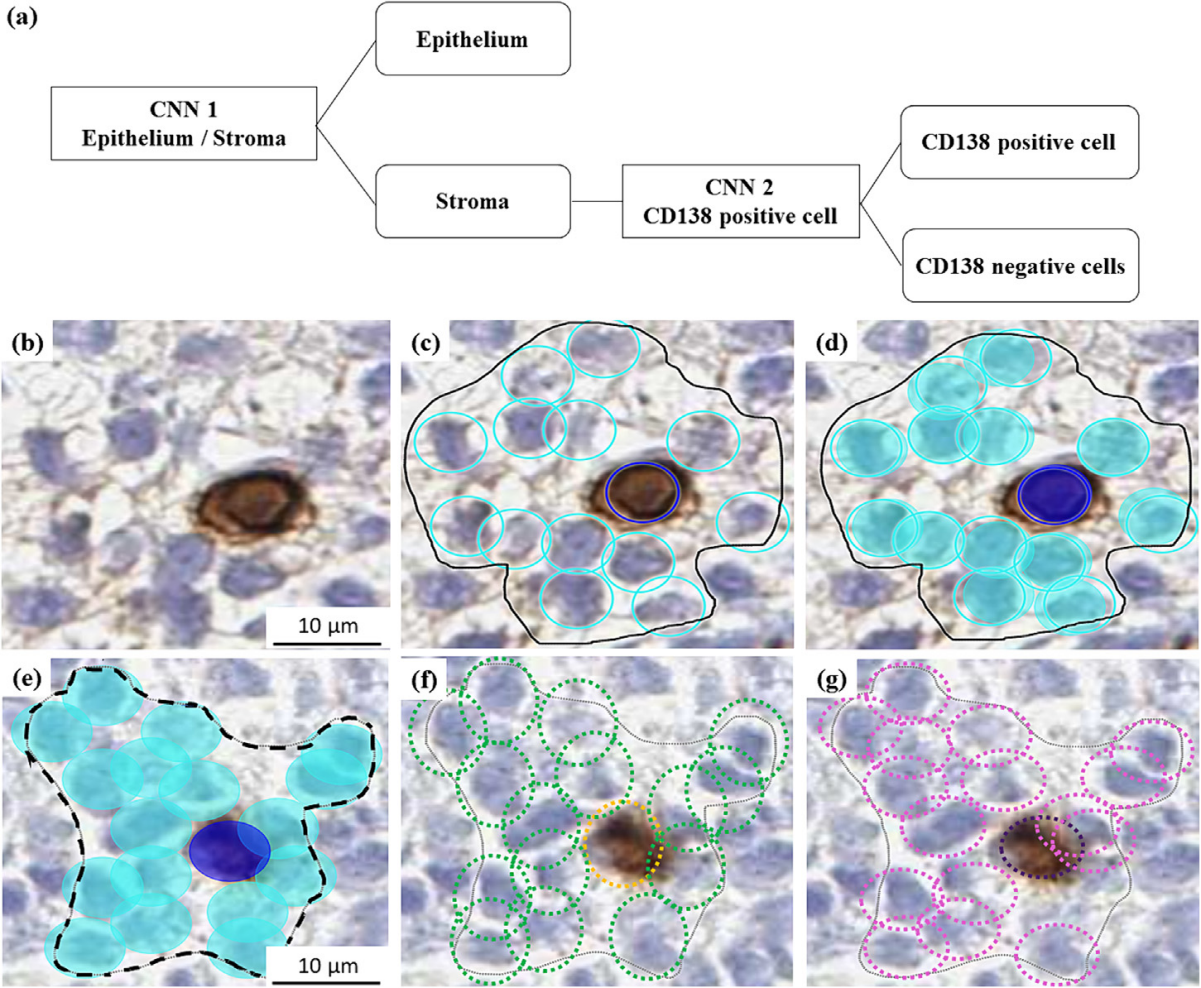
Schematic overview of the AI algorithm and examples of training and validation. (a) The structure of the convolutional neural networks (CNNs) model includes CNN1 for the regional layer and CNN2 for the object layer. (b)–(d) Examples of the AI algorithm training, which was conducted through manual annotation. Only the areas within the regions of interest (ROIs) outlined as solid black lines were considered in the training. (b) original image, (c) manually annotated cells. (d) The AI algorithm training result. CD138− cells were marked cyan, while CD138+ cells were marked dark blue. (e)–(g) The training validation of the AI algorithm, where the validation regions were marked as black dotted line. (e) Analyzed images by the AI algorithm (CD138− cells marked cyan, CD138+ cells marked dark blue), (f) the validation from validator 1 (CD138- cells marked green, CD138+ cells marked yellow), (g) the validation from validator 2 (CD138− cells marked pink, CD138+ cells marked purple). (For interpretation of the references to color in this figure legend, the reader is referred to the web version of this article.)

Our previous AI algorithm, AINO, was modified and trained to detect CD138+ plasma cells in the endometrial stroma.^16^ Our current AI algorithm was trained using morphological features derived from 150 WSIs across two layers: the regional layer and the object layer. All training was performed on regions of interest (ROIs) drawn to remove noise and background interference that could affect the analysis. The regional layers were trained to segment the epithelium and stroma, followed by training of the object layers to differentiate CD138− and CD138+ cells within the stroma. The training set consisted of a 28,363 mm^2^ region and 7345 object layers (6942 CD138- cells and 403 CD138+ cells). The algorithmic structure of the model and the training process are shown in Fig. 2a–d.

### AI algorithm validation and analysis

The accuracy and reliability of the AI algorithm were assessed through a two-step validation by the three external validators (O.L., J.K., and H.M.) who did not take part in the AI algorithm training to avoid biased decisions.

The training validation was performed in 18 WSIs not used for the model training. A total of 76 ROIs (36 regional and 40 object layers) were selected in each cycle phase and study group. The validators (O.L. and J.K.) annotated all the compartments, and the annotation was compared to the AI algorithm (Fig. 2e–g). The error indices were automatically calculated in the AI platform.^17^ Briefly, the total error indicates a sum of false negative (FN) and false positive (FP) predictions, precision is measured by dividing the true positive (TP) by all positives, sensitivity is measured by dividing the TP by the sum of the TP and the FN, and the F1 score is measured by the harmonic mean of precision and sensitivity. Furthermore, specificity is determined by dividing the true negative (TN) by the sum of TN and FP, while accuracy is computed by dividing the sum of TP and TN by the sum of TP, FP, FN, and TN.

In the performance validation, the validators (O.L., J.K., and H.M.) manually counted the number of stromal CD138+ cells in 10 randomly selected high-power fields (HPFs) on each slide for a total of 10 slides. Since a 1-mm^2^ unit area equals four HPFs,^29^ the cell counts from AI analysis were divided by 4 to convert to cells/HPF format. These converted data were compared to the median number of CD138+ cells determined by the validators.

After the validation, the analysis for 182 WSIs (i.e., 71 control, 82 PCOS, and 29 RIF samples) was performed. CD138+ cell percentages were calculated manually by dividing the total number of CD138+ cells by the sum of CD138− and CD138+ cells in all ROIs within a WSI and then multiplying by 100.

### Statistical analysis

Statistical analyses were conducted using IBM SPSS Statistics v28 (IBM Corp., Armonk, NY, United States), and visualization was created using GraphPad Prism (version 9.3.0) and RStudio (version 2022.12.0.353 with R version 4.2.2). For the women who had provided more than one sample, the CD138+ cell percentages and clinical features were counted as individual samples in each cycle phase. The cell percentages based on PCOS diagnosis and cycle phases were analyzed by the mixed-model analysis of variance (ANOVA), in which the total variance is divided into the within-group variance and the between-group variance. The continuous variables were tested by a Mann–Whitney *U* test for paired tests and a Kruskal–Wallis test for multiple comparisons depending on the data distribution. Statistical significance was defined as *p* < 0.05. An intraclass correlation (ICC) estimate was calculated using a two-way mixed effects model with an absolute agreement model, with ICC values interpreted as poor (<0.5), moderate (0.5–0.75), good (0.75–0.9), and excellent (>0.9) reliability.^30^ Correlations between the CD138+ plasma cell percentages and clinical characteristics were calculated using Spearman correlations, considering data availability and distribution.

## Results

### Training and validating the AI-algorithm

The final training errors for the regional layers (i.e., epithelium and stroma) and the object layers (i.e., CD138−/+ stromal cell) were 2.28% and 6.15% (CD138+ cells 3.23%, CD138− cells 6.32%), respectively (Table 2). In the training validation, median values of precision, sensitivity, F1 score, and specificity were calculated from 18 WSIs between pathologists (Supplemental Fig. S1a). All verification statistics for CD138+ cells were 100%, indicating that the decisions made by the pathologists and the AI algorithm were in complete agreement. For the performance validation, we compared the number of CD138+ cells quantified by the pathologists and the AI analysis in 10 slides (Supplemental Fig. S1b). The two evaluation methods showed excellent accuracy (ICC, 0.76; 95% confidence intervals, 0.36 to 0.93; *p* = 0.002) and a positive correlation (Spearman's rank correlation coefficient, 0.79; *p* < 0.01). The interobserver variability between pathologists indicated good to excellent reliability for training validation (ICC, 0.86–0.93) and good reliability for performance validation (ICC, 0.82) (Supplemental Table S2). Therefore, the AI algorithm demonstrated sufficient functionality in identifying the target plasma cells.

**Table 2:** The AI algorithm training results.

(%)	CNN 1	Epithelium	Stroma	CNN 2	CD138+	CD138−
Total error	2.28	15.20	1.04	6.15	3.23	6.32
Precision	99.17	92.86	99.82	99.45	100	99.42
Sensitivity	98.46	91.86	99.14	94.36	96.77	94.22
F1 score	98.81	92.36	99.48	96.84	98.36	96.75
Specificity	99.60	92.94	99.82	99.48	100	99.45
Accuracy	99.23	92.40	99.23	96.92	98.39	96.84

CNN 1 encompasses epithelium and stroma, while CNN2 encompasses CD138+ and CD138− cells. The total error (FP + FN), precision (TP/[TP + FP]), sensitivity (TP/[TP + FN]), F1 score (2 × Precision × Sensitivity/[Precision + Sensitivity]), specificity (TN/[TN + FP]), and accuracy ([TP + TN]/[TP + TN + FP + FN]) percentages were calculated to evaluate the AI model training.

CNN (convolutional neural network), false positive (FP), false negative (FN), true positive (TP), true negative (TN).

### Endometrial stromal CD138+ plasma cell percentages by cycle phase and PCOS status

**Fig. 3:**
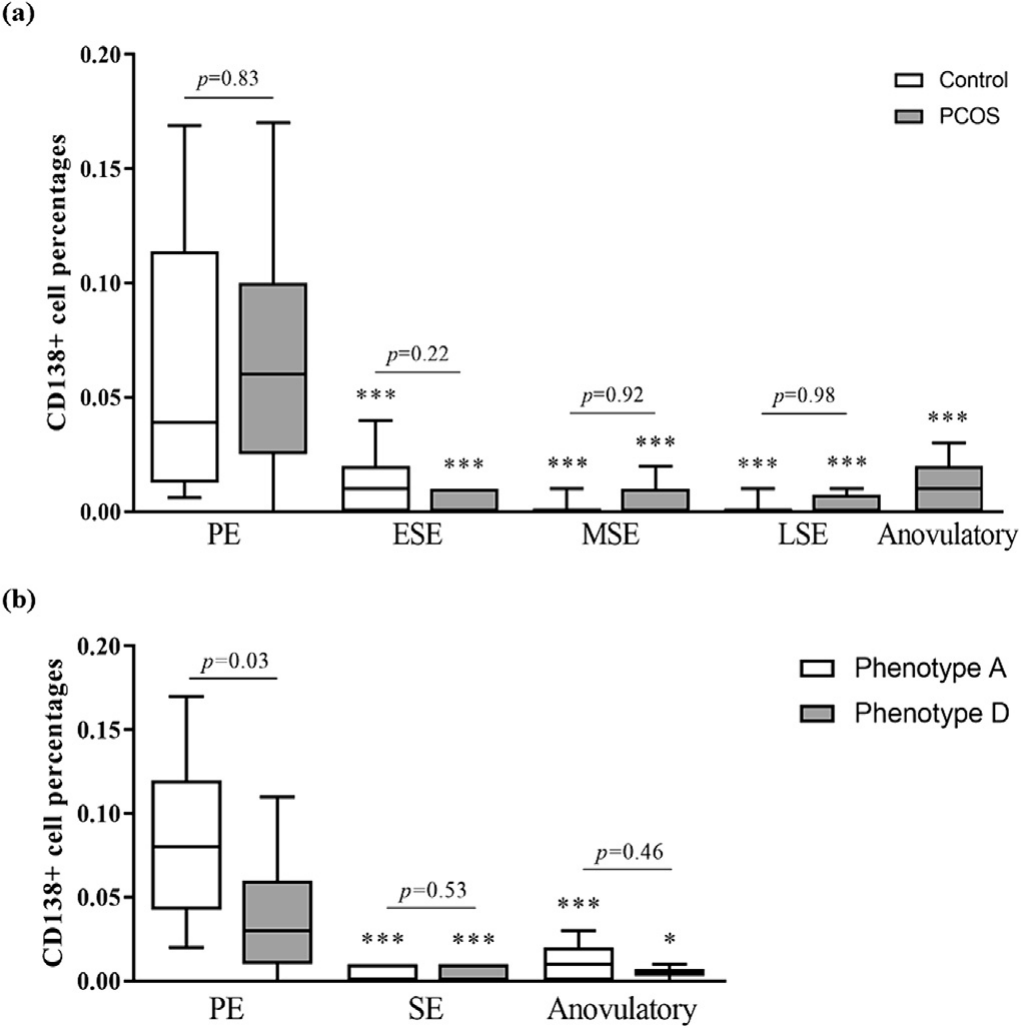
Stromal CD138+ plasma cell percentages across PCOS status and cycle phases. Stromal CD138+ cell percentages for: (a) controls (12 PE, 15 ESE, 25 MSE, 19 LSE) and PCOS cases (21 PE, 14 ESE, 21 MSE, 16 LSE, 11 Anovulatory) and (b) different PCOS phenotypes (Phenotype A; *n* = 12 PE, *n* = 26 SE, *n* = 8 Anovulatory, Phenotype D; *n* = 9 PE, *n* = 16 SE, *n* = 2 Anovulatory). Phenotype C was excluded due to the small sample size. The box indicates the interquartile range, the middle line represents the median, and the whiskers show the min–max range. ***p < 0.001 compared to the PE samples. The statistical differences were calculated by the mixed-model ANOVA. PE (proliferative phase), ESE (early secretory phase), MSE (mid-secretory phase), LSE (late secretory phase), SE (secretory phase).

The AI analyzed 71 controls and 82 PCOS slide images, grouped by PCOS diagnosis/phenotypes and menstrual cycle phases. CD138+ cell percentages showed a significant decrease from the PE to the SE, regardless of PCOS diagnosis (*p* < 0.001) (Fig. 3a). Furthermore, we observed no distinctions in the CD138+ cell proportion between control and PCOS cases during the same cycle phase (*p*_PE_ = 0.83, *p*_ESE_ = 0.22, *p*_MSE_ = 0.92, *p*_LSE_ = 0.98). Interestingly, anovulatory PCOS cases exhibited similar CD138+ cell percentages than the PCOS SE samples (*p* > 0.58) but markedly lower than PCOS PE samples (*p* < 0.001). In the phenotype comparisons, we pooled the three different SEs into one SE (i.e., ESE + MSE + LSE) to simplify the comparisons between the cycle phases and enhance the power of statistical analysis. Phenotype A cases showed significantly higher CD138+ cell percentages compared to phenotype D cases in the PE (*p* < 0.001) (Fig. 3b).

### Correlations between endometrial stromal CD138+ plasma cell percentages and clinical characteristics in women with PCOS and non-PCOS controls

To validate the correspondence between the AI analysis and menstrual cycle-related traits and elucidate factors contributing to increased CD138+ cell percentages in the PE, we analyzed correlations between the plasma cell percentages and physiological features (Table 3). Control SE samples showed negative correlations with P4 (r^2^ = −0.43, *p* = 0.02) and positive correlations with FAI and LH (FAI; r^2^ = 0.45, *p* < 0.001, LH; r^2^ = 0.41, *p* = 0.02). In contrast, PCOS SE cases exhibited positive correlations with testosterone (r^2^ = 0.32, *p* = 0.02) and AMH (r^2^ = 0.30, *p* = 0.03). Meanwhile, we did not observe any significant correlations in the PCOS anovulatory group.

**Table 3:** Correlations between the CD138+ cell percentages and endometrium thickness and hormonal values.

	CD138+ percentages	Endometrial thickness (mm)	P4 (nmol/L)	Testosterone (nmol/L)	FAI	FSH (IU/L)	LH (IU/L)	AMH (ng/ml)
*Control*								
Control PE (*n* = 12)	0.04 [0.01, 0.11]	5.25 [4.43, 5.78] (−0.16)	14.60 [1.34, 33.34] (−0.035)	1.01 [0.80, 1.38] (0.06)	2.20 [1.50, 2.76] (0.13)	7.41 [6.68, 8.75] (0.33)	7.69 [6.70, 9.23] (0.19)	2.28 [1.22, 4.64] (−0.40)
Control SE (*n* = 59)	0.00 [0.00, 0.01]	9.80 [8.40, 11.30] (0.21)	25.35 [4.78, 41.61] (−0.43**)	1.17 [0.95, 1.65] (−0.11)	2.00 [1.24, 2.93] (0.45***)	3.68 [2.61, 4.69] (0.15)	7.64 [4.82, 10.34] (0.41**)	2.25 [1.35, 4.48] (0.06)
*PCOS*								
PCOS PE (*n* = 21)	0.06 [0.03, 0.10]	5.35 [4.53, 6.03] (0.02)	11.21 [0.25, 48.12] (0.33)	1.00 [0.83, 1.77] (−0.02)	2.64 [1.87, 3.22] (0.30)	6.79 [6.13, 8.63] (−0.52*)	9.72 [8.47, 11.27] (−0.03)	5.27 [2.67, 7.40] (0.24)
PCOS SE (*n* = 50)	0.00 [0.00, 0.01]	9.70 [8.00, 10.50] (−0.05)	22.49 [0.37, 41.06] (−0.24)	1.33 [1.00, 1.68] (0.32*)	2.51 [1.61, 3.34] (0.09)	3.57 [2.62, 5.03] (−0.03)	8.97 [5.19, 13.20] (0.14)	3.68 [2.50, 5.83] (0.30*)
PCOS Anovulatory (*n* = 11)	0.01 [0.00, 0.02]	5.60 [5.00, 8.95] (0.05)	0.29 [0.26, 0.33] (−0.17)	1.65 [1.24, 2.49] (−0.47)	6.42 [3.34, 11.82] (−0.05)	6.59 [5.65, 7.59] (0.36)	16.46 [10.69, 20.05] (0.03)	9.08 [5.01, 12.80] (0.02)

The CD138 cell percentages and clinical characteristics presented as median with interquartile range [Q1;Q3]. The correlations are presented in brackets. *p* values were determined using log-transformed values of sex-hormone-related characteristics for the skewed data distribution. * *p* < 0.05, ** *p* < 0.01, and *** *p* < 0.001.

P4 (progesterone), FAI (free androgen index), FSH (follicle-stimulating hormone), LH (luteinizing hormone), AMH (anti-Müllerian hormone).

### Endometrial stromal CD138+ plasma cell percentages by receptivity in RIF samples

**Fig. 4:**
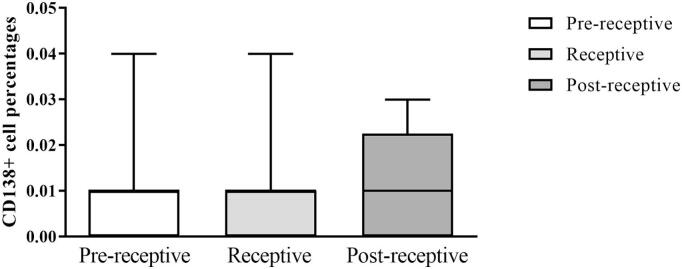
Stromal CD138+ plasma cell percentages stratified by endometrial receptivity. The CD138+ cell percentage comparisons of RIF patient samples (*n* = 9 RIF pre-receptive, *n* = 10 RIF receptive, *n* = 10 RIF post-receptive). The box indicates the interquartile range, the middle line represents the median, and the whiskers show the min–max range. The Kruskal–Wallis test did not reveal any significant differences between the groups.

Next, we investigated potential variations in CD138+ cell percentages across different endometrial receptivity statuses defined by gene expression. We used the endometrial samples that were timed after P4 administration at P4 + 5 days, which is considered the WOI and common time for embryo transfer in assisted reproductive technology. We did not observe any differences in CD138+ cell percentages based on the receptivity among RIF samples (*p* = 0.81) (Fig. 4).

## Discussion

In this study, we developed an AI algorithm to quantify CD138+ plasma cells and investigate the factors influencing CD138+ cell aggregation in the human endometrium without any other signs of CE. We verified that the AI algorithm performed with high precision and accuracy through a two-step validation process. Additionally, the analysis results align with previous research indicating a higher number of CD138+ plasma cells in the PE endometrium than in the SE endometrium, supporting the reliability of the AI model.^8^^,^^31^ As a novel finding, anovulatory PCOS samples showed significantly lower CD138+ cell percentages compared to the PE PCOS samples, and phenotype A PCOS samples exhibited higher CD138+ cell percentages in the PE than phenotype D PCOS samples. On the other hand, the cell percentages did not vary based on endometrial receptivity in RIF samples.

The introduction of WSIs represents a transition in histological examination from glass slides viewed under a microscope to digital images displayed on computer monitors.^32^ The adoption of WSIs has overcome practical constraints inherent in traditional histology. For instance, storing WSIs digitally prevents loss of information in tissue slides caused by physical damage or degradation over time.^32^ Digitalized WSIs enable remote communication, thereby circumventing limitations related to time and geographic location.^32^, ^33^, ^34^ AI has revolutionized the rapid analysis of a large number of WSIs at high pixel resolution, overcoming challenges, errors, or difficulties inherent in human eye-driven assessment.^32^^,^^35^ Furthermore, AI has the potential to reduce both intra- and interobserver variations, which can result in diagnostic inconsistencies and potentially undermine the quality of patient care by eliminating the subjective nature of observation.^13^^,^^26^ Therefore, AI can reduce the workload of pathologists and improve the reliability and reproducibility of histopathological analysis, contributing to its widespread application across various research fields.^13^^,^^36^^,^^37^ However, the application of CNN algorithms in uterine studies has predominantly focused on cancer research.^18^^,^^38^ To our knowledge, our study is the first to target endometrial CD138+ plasma cells in a whole view of slides employing a cloud-based CNN platform.^15^^,^^17^^,^^26^ Remarkably, we were able to analyze 182 WSIs within an hour, a task that would be unfeasible for a pathologist to complete alone. However, we encountered specific challenges with certain endometrial features during the training and analysis steps, particularly with pre-decidual structures in the stroma. There were instances where the AI model mistakenly identified these structures as epithelia, thus disregarding the stromal cells in those regions. Nevertheless, based on comparisons with previous studies using the same AI platform, we evaluated the overall performance of our AI model as excellent.^14^^,^^15^^,^^17^

The higher CD138+ cell percentages observed in the PE compared to the SE may be due to the estradiol-mediated recruitment of plasma cell progenitors from the systemic circulation into the endometrial mucosa, supported by a previous study in mice.^39^ Furthermore, as the endometrium thickens toward the SE, biopsies taken during this phase will likely sample predominantly from the superficial layers, potentially leading to lower CD138+ cell percentages relative to unstained stromal cells.^31^ These hypotheses do not, however, fully account for our results of lower CD138+ cell percentages in anovulatory and amenorrhoeic PCOS samples compared to those in the PE PCOS samples, as the endometrial thickness was comparable. Considering that anovulatory endometrium is characterized by increased expression of estrogen receptors, heightened estrogen sensitivity, and greater estrogen exposure,^20^^,^^40^ further research with a larger sample size is necessary to explore the impact of E2 on the presence of CD138+ cells throughout the menstrual cycle and in the anovulatory PCOS endometrium. In addition, higher CD138+ percentages in phenotype A PCOS compared to phenotype D, particularly at the beginning of a menstrual cycle (cycle days 6–8), suggest a possible influence of HA on the recruitment of CD138+ cells, warranting further investigation.

Our findings showed that the receptivity status on day 5 of HRT did not influence the CD138+ cell accumulation. Given that previous findings suggest an increased prevalence of endometrial inflammation (or even CE) in infertile women based on CD138+ plasma cell aggregation,^7^^,^^8^ future studies should consider larger sample sizes and incorporate prospective study designs, including women undergoing IVF treatment with natural menstrual cycles or stimulated cycles versus HRT cycles.

The main strength of our study lies in the remarkable efficiency and reliability of our AI algorithm, which can swiftly identify stromal CD138+ plasma cells within a WSI in just a few seconds. Furthermore, our study benefits from a unique and well-characterized human endometrial tissue sample set, enabling us to cover a wide range of endometrial conditions: (i) across the menstrual cycle phases, (ii) individuals with PCOS or RIF diagnoses, (iii) both ovulatory and anovulatory statuses, and (iv) different endometrial receptivity statuses.

As for limitations, we lacked confirmed CE cases to serve as positive controls, although our primary focus was not on diagnosing overt endometritis. Additionally, despite more severe hormonal derangement in anovulatory PCOS cases, we found no significant differences in CD138+ cell percentages compared to PCOS SE samples nor significant correlations between CD138+ plasma cell percentages and clinical characteristics. This may be attributed to the limited number of anovulatory PCOS samples. Detailed clinical data for the RIF patients, particularly regarding serum sample analysis, were unavailable, hindering exploration of associations between CD138+ cell percentages and clinical characteristics, and further limiting our analysis due to the absence of non-RIF controls under HRT. Lastly, we could not use serum estrogen levels since they were below the detection threshold by LC-MS in both the control and PCOS populations during a single run processing for a panel of steroids. Despite these limitations, our study highlights the significant potential of AI technology in addressing current clinical and practical challenges associated with the detection of endometrial immune cells.

## Conclusion

Our findings underscore the precision and potential of the AI algorithm in identifying endometrial CD138+ plasma cells, aligning with physiological characteristics. The algorithm reveals that the occurrence of CD138+ is influenced by PCOS phenotypes and menstrual cycle phases, whereas receptivity status in RIF did not seem to play a role. The AI algorithm provides unique advantages, such as swift inspection of WSIs, sparing the valuable time of trained pathologists, and reliable, reproducible analysis. This highlights the practicality of incorporating AI technology into clinical decision-making, particularly for understanding dynamics related to the endometrial cycle phase and various reproductive disorders.

## Ethics statement

This study was approved by The Regional Ethics Committee of the Northern Ostrobothnia Hospital District, Finland (65/2017), and the Research Ethics Committee of the University of Tartu, Estonia (340 T-12). All experiments were performed in accordance with relevant guidelines/regulations in line with the Declaration of Helsinki.

## Author contributions

T.T.P., A.S., R.K.A., and S.L. designed the study. T.T.P., A.S., R.K.A., E.K.K., and M.S. contributed to sample collection. A.A., K.K., and M.S. were involved in sample processing. O.L., J.A.K., and H.M. performed the validation. S.L. and E.K.K. performed image analysis. S.L. and R.K.A. performed the statistical analysis. All authors revised and approved the final version.

## Funding

This research was funded by the 10.13039/501100002341Academy of Finland, the Sigrid Jusélius Foundation, 10.13039/501100009708Novo Nordisk Foundation, and the European Union's Horizon 2020 research and innovation programme under the Marie Sklodowska-Curie grant (MATER, grant no. 813707). This research was also funded by the 10.13039/501100002301Estonian Research Council (grant no. PRG1076), Horizon 2020 innovation grant (ERIN, grant no. EU952516), 10.13039/501100006598Enterprise Estonia (grant no. EU48695), and MSCA-RISE-2020 project (TRENDO, grant no. 101008193). The funders did not participate in any processes of the study.

## Declaration of competing interest

The authors declare that the research was conducted in the absence of any commercial or financial relationships that could be construed as a potential conflict of interest.

## Data Availability

The datasets generated during and/or analyzed during the current study are not publicly available due to sensitivity of the health data. Non-personal data can be requested from the corresponding author.
