# Supplementary material for: AI-algorithm training and validation for identification of endometrial CD138+ cells in infertility-associated conditions; polycystic ovary syndrome (PCOS) and recurrent implantation failure (RIF)
Source: J Pathol Inform. 2024 Apr 29;15:100380. doi: 10.1016/j.jpi.2024.100380 (PMC11140811; doi:10.1016/j.jpi.2024.100380)
Supplement: Supplementary file 1 — Supplementary material [file mmc1.docx]

Supporting information

**Supplemental Figure S1. The AI algorithm validation result.**

**Supplemental Table S1. Baseline characteristics of PCOS subjects by phenotypes**

**Supplemental Table S2. Interobserver variability in the AI algorithm validation**

**Supplemental Figure S1. The AI algorithm validation result.**

The AI algorithm was validated in two stages: (a) training validation and (b) performance validation. (a) The heatmap, generated by calculating the median between the two validators, presents the agreements between the AI algorithm and the validators. (b) The number of CD138+ cells per high power field (HPF) from manual counting was compared to the AI analysis results. The area unit (mm2) was converted to HPF (1 HPF=0.25 mm^2^). Validation values were determined by calculating the median between the three validators.
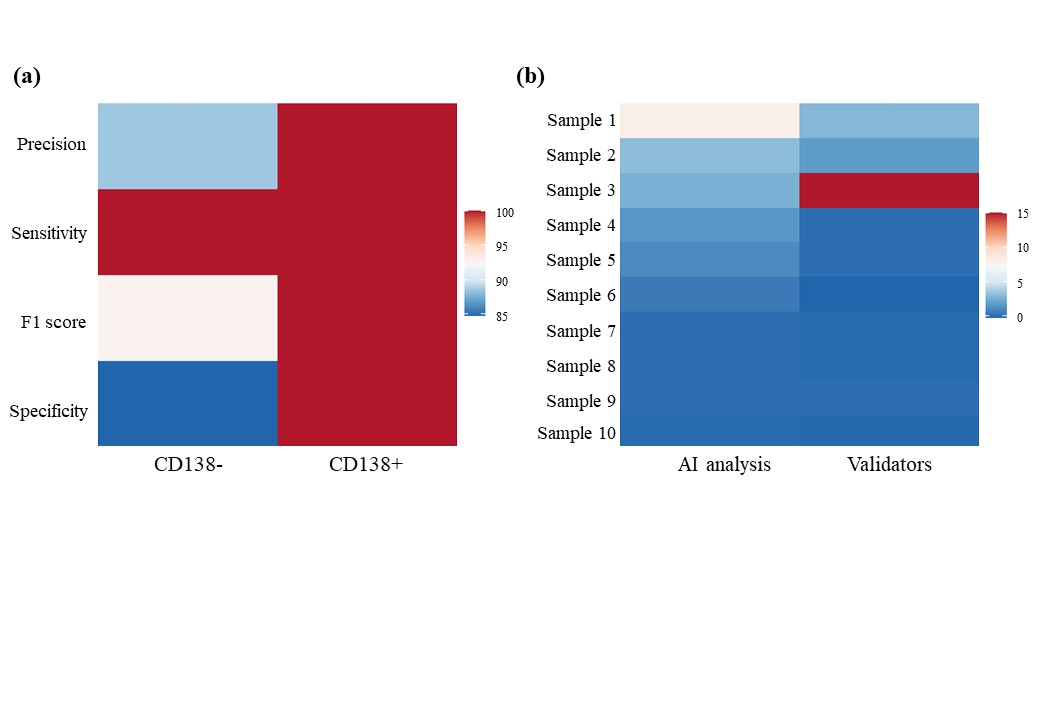


| **Supplementary Table S1. Baseline characteristics of PCOS subjects by phenotypes** | | | |
| --- | --- | --- | --- |
|  | Phenotype A | Phenotype D | FDR |
| Age, years old | 33.00 [29.00;38.00] (35) | 35.00 [30.00;37.00] (19) | 0.88 |
| BMI, kg/m2 | 26.60 [23.70;34.60] (35) | 27.70 [22.70;30.10] (19) | 0.68 |
| WHR | 0.84 [0.80;0.90] (35) | 0.79 [0.76;0.84] (19) | 0.06 |
| Fasting glucose, mmol/L | 5.30 [5.10;5.80] (35) | 5.20 [4.90;5.50] (19) | 0.45 |
| Fasting insulin, mU/I | 9.90 [6.80;16.90] (34) | 6.50 [4.60;8.90] (19) | **0.01** |
| HOMA-IR index | 2.24 [1.57;4.36] (35) | 1.47 [1.02;2.13] (19) | **0.04** |
| Testosterone, nmol/L | 1.40 [1.23;2.22] (35) | 1.19 [0,96;1.42] (19) | **0.04** |
| AMH, ng/mlL | 5.51 [4.16;8.22] (35) | 3.95 [2.27;5.01] (19) | **0.03** |
| Clinical characteristics are presented as median with interquartile range [Q1;Q3]. The number of subjects is indicated in parentheses. *p*-value was determined by the Mann-Whitney *U*-test with the Benjamini-Hochberg FDR adjustment, and bold values represent FDR<0.05.  BMI (body mass index), WHR (Waist-hip ratio), HOMA-IR (homeostatic model assessment for insulin resistance), AMH (Anti-Müllerian hormone) | | | |

| **Supplementary Table S2. Interobserver variability in the AI algorithm validation** | | | | |
| --- | --- | --- | --- | --- |
| ICC (95% CI) | Epithelium | Stroma | CD138- cell | CD138+ cell |
| Training  between two pathologists | 0.933*******  (0.869,0.966) | 0.927*******  (0.855,0.963) | 0.858*******  (0.719,0.927) | - |
| Performance  between three pathologists |  |  |  | 0.820******  (0.486,0.951) |
| ** *p* <0.01, *** *p* <0.001  ICC (intraclass correlation coefficient), CI (confidence interval) | | | | |
